# Intravesical antimicrobial administration for urinary tract infections via a dedicated catheter

**DOI:** 10.1002/bcp.70124

**Published:** 2025-06-10

**Authors:** John Posner

**Affiliations:** ^1^ Institute of Pharmaceutical Science King's College London London UK

The European Association of Urology (EAU) defines recurrent urinary tract infection (rUTI) as ‘two or more episodes of symptomatic UTI within 6 months, or three or more within 12 months’. Guidelines available for management of rUTIs in women have been reviewed and summarized.^1^ Most recommend treatment with short courses of antibiotics for acute episodes and prophylaxis with continuous or post‐coital antibiotics, increased fluid intake and topical vaginal oestrogen for postmenopausal women. Strategies to prevent rUTIs in particular groups of patients include use of cranberry products containing proanthocyanidin and oral methenamine hippurate or mandelate. Immunoprophylaxis with oral OM‐89, a bacterial lysate extract from *Escherichia coli* that is thought to stimulate the host immune system, is also generally supported.^2^

Complicated UTIs, as distinct from recurrent UTIs, include those related to pregnancy, anatomical or functional abnormalities of the urinary tract, indwelling urinary catheters, renal disease and other immunocompromising diseases such as diabetes. Patients with chronic neuropathic conditions such as spinal injury or multiple sclerosis are at particular risk of recurrent infections and septicaemia. Use of indwelling Foley catheters, particularly in the elderly and patients with neuropathy, is widely practised, but they act as a source of repeated infection.

Treatment failure and recurrence of infections associated with increasing development of antimicrobial resistance (AMR) may partly be attributable to the way in which antibiotics are used including overuse, misuse and suboptimal infection prevention practices.^3^ Whether administered by the oral or intravenous route only a fraction of the administered dose actually reaches the urine and bladder urothelium in concentrations that exceed the minimum inhibitory concentration (MIC) for adequate duration. While bactericidal efficacy in serum for systemic infections is dependent on the ratios between plasma C_max_ and AUC to the MIC,^4^ these data are far less applicable to infection localized to the bladder. Furthermore, the drug that does reach the bladder is diluted by urine, thereby reducing the probability of attaining and maintaining bactericidal concentrations. The importance of achieving and testing antimicrobial sensitivity based on urinary rather than serum concentrations is not new and was the subject of a paper published over 50 years ago.^5^ Adverse effects are also a major consideration for some antibiotics administered by a systemic route.

These serious and common shortcomings in the treatment and prevention of cystitis with antibiotics may at least be partially overcome by direct intravesical administration (IVA) after voiding urine. Importantly, the permeability of the urothelium is very low so that drug permeation into the systemic circulation and the gut should be negligible.^6^ IVA for treatment of bladder conditions is not novel. Intravesicular Bacillus Calmette–Guerin (BCG) has been used for many years for treatment of non‐muscle invasive bladder cancer as have chemotherapies such as gemcitabine and mitomycin. Intravesical interferon gene therapy with adenoviral vector‐based nadofaragene firadenovec has also been approved by the FDA for adult patients with high‐risk BCG‐resistant, non‐muscle invasive bladder cancer. Severe cases of irritable bladder can also be treated with intravesical botulinum toxin.

The conduct of adequately powered, high‐quality, prospective clinical trials of intravesical antimicrobials for cystitis has been limited to date and does not appear to have been of interest to drug regulatory authorities. In a review of five prophylactic and six treatment studies involving intravesical gentamicin, neomycin with polymyxin, neomycin or colistin a good reduction in symptomatic UTI was seen in 78% (120/168) of patients in the prophylaxis group and 88% (103/117) in the treatment groups. Treatments were tolerated well with few adverse effects, and discontinuation rates were less than 10%.^7^ The authors concluded that IVA appeared to be safe and effective for both prophylaxis and treatment of rUTIs.

In a prospective study of intravesical instillations of gentamycin to 63 adult patients with rUTIs caused by multidrug resistant pathogens, overnight intravesical instillations of gentamicin were administered for 6 months.^8^ The mean number of UTIs was reduced from 4.8 to 1.0 during treatment and the resistance rate of the uropathogens decreased from 78% to 23%. No systemic absorption or clinically relevant side effects were observed.

To summarize, of the antibiotics available for IVA, there are encouraging data with gentamycin, but it is clear that randomized, controlled clinical trials are needed to assess the acceptability and effectiveness of IVA with different antibiotics. IVA of non‐antibiotic prophylactic agents has recently been reviewed.^9^ Instillation of hyaluronic acid alone and combined with chondroitin sulphate targets adherence of bacteria to the bladder mucosa, which is attributable to a damaged glycosaminoglycan (GAG) layer. Recurrence of cystitis, urinary symptoms, quality of life and cystometric capacity have been shown to be superior to antibiotic prophylaxis with substantially longer times to recurrence.

The paucity of clinical trials of antibiotics or prophylactic agents by the intravesical route may partly be explained by the lack of suitable catheters. A catheter for dedicated intravesicular use that can be inserted into the urethra by the patient or carer without significant risk of introducing bacteria is of critical importance for treatment of cystitis. A single‐use disposable narrow‐gauge catheter would be appropriate for safe and painless IVA after application of antiseptic gel or possibly a rapidly acting prophylactic agent. Instillation of the antibiotic or other treatment should be through a lumen separate from that for urine drainage using an easily‐attached pre‐filled syringe with valves to prevent backflow of both urine and therapeutic agent.

Evaluation of treatments with such a device should proceed with high‐quality randomized, controlled clinical trials using different antibiotics or other agents and a range of doses determined from the MIC in urine. The instillation should take place after emptying the bladder and should be retained in the bladder for at least an hour to maximize the chance of achieving eradication of the infection. The number of administrations will depend on the indication. As well as severity of symptoms including pyrexia, trials should compare rates of the four patterns of bacteriuria response: (i) cure, (ii) persistence of the same bacterium after treatment, (iii) relapse with the same microorganism after cessation of treatment and (iv) reinfection, that is, usually with a different bacterial species after initial successful sterilization of the urine.^10^ Suitable populations for such trials should include various groups of patients with recurrent cystitis and those with complicating risk factors such as diabetes and neuropathic conditions including multiple sclerosis and spinal cord injuries. Such topical therapy has the potential to reduce the frequency and severity of recurrent UTIs and, if successful, could reduce the ever‐present and increasing risk of AMR particularly in the elderly population.
